# Investigating the structural properties and in vitro digestion of rice flours

**DOI:** 10.1002/fsn3.2225

**Published:** 2021-03-13

**Authors:** Muhammad Adil Farooq, Mian Anjum Murtaza, Rana Muhammad Aadil, Rizwan Arshad, Abdul Rahaman, Rabia Siddique, Sadia Hassan, Hafiz Muhammad Saleem Akhtar, Muhammad Faisal Manzoor, Emad Karrar, Amjad Ali, Ahsan Ul Haq

**Affiliations:** ^1^ School of Food and Biological Engineering Jiangsu University Zhenjiang China; ^2^ Institute of Food Science and Nutrition University of Sargodha Sargodha Pakistan; ^3^ National Institute of Food Science and Technology University of Agriculture Faisalabad Pakistan; ^4^ University Institute of Diet and Nutritional Sciences University of Lahore, Chenab Campus Gujrat Pakistan; ^5^ School of Food Science and Engineering South China University of Technology Guangzhou China; ^6^ Department of Chemistry Government College University Faisalabad Faisalabad Pakistan; ^7^ Department of Nutritional Sciences Government College Women University Faisalabad Pakistan; ^8^ College of Food Science and Technology Nanjing Agriculture University Nanjing China; ^9^ Department of Food Engineering and Technology Faculty of Engineering and Technology University of Gezira Wad Medani Sudan; ^10^ Department of Agriculture and Food Technology Karakoram International University Gilgit Pakistan; ^11^ Department of Forestry and Range Management University of Agriculture Faisalabad Pakistan

**Keywords:** amylose content, digestibility, gelatinization, rice flour, X‐ray diffraction

## Abstract

The physicochemical properties, swelling power, solubility, and digestibility of flour from four rice varieties (black, brown, white, and waxy rice flour) were analyzed. The results showed that the black and brown rice had high‐amylose percentage (21.8% and 20.5%), a relatively low percentage of starch content (68.1% and 79.1%), and lower swelling power (6.6% and 7.6%) and solubility (13.5% and 15.7%), respectively. Waxy rice flour attributed to lower gelatinization temperatures and higher enthalpy values. Meanwhile, the brown, black, and white rice showed higher gelatinization temperature and lower enthalpy value. The black and brown rice flour exhibited lower pasting and viscosity values as compared to waxy rice flour. The results showed that all rice flour had an A‐type X‐ray diffraction pattern, and after cooking all rice flour showed V‐type polymorphs except waxy rice flour. Brown and black rice flour after cooking have lower digestion rate than white rice and waxy rice flour, probably due to its lower expansion and solubility rates, and higher gelatinization temperature.

## Graphical abstract

*Rosa Roxburghii* seed oil was of high quality in terms of its high amount of unsaturated fatty acids. Heat treatment of *R. roxburghii* seed oil showed an increase in the relative percentage of linoleic acid and linolenic acid isomers with increasing temperature and time. The formation of linoleic acid and linolenic acid isomers followed a zero order reaction.

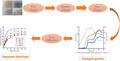

## 1 INTRODUCTION

Rice is a major source of energy that is consumed as a staple food throughout the world, especially in Asia. As rice flour is gluten‐free, therefore, it is recommended to make gluten‐free food products (Mbanjo et al., 2020). Several commercial gluten‐free products such as bread, noodles, and cakes are made from white rice due to effective economical raw material (Mun & Shin, 2018). The consumption of white rice and its products increases the risks of diabetes and metabolic diseases (An et al., 2016; Thiranusornkij et al., 2018). As the diabetic's prevalence is increasing in the world, therefore, the glycemic index needs to be reduced in rice‐based food products by screening the rice varieties with low starch digestibility (Klunklin & Savage, 2018; Wang et al., 2017).

In recent times, dark‐colored rice has developed as a potential functional food due to its nutritional and phytochemical composition (anthocyanins, carotenoids, flavones, flavonols, and γ‐oryzanols) (Pereira‐Caro et al., 2013; Thiranusornkij et al., 2018). Brown rice contains a higher amount of nutrients than white rice due to the presence of higher contents of protein, minerals, and dietary fiber, which show a positive impact on human health such as reduced risks of cancer, cardiovascular disease, and type 2 diabetes (Liu, 2007). Similarly, black rice is consumed in Asia, particularly in China due to its nutritional value, color, and unique flavor aspects. Anthocyanin pigments are responsible for black color. Black rice is used in many food products as a coloring agent and functional food due to its high phenolic contents (Bolea & Vizireanu, 2017). Moreover, black rice decreases the risk of diseases associated with chronic inflammation and acts as an anti‐allergic and anti‐inflammatory food component (Dhital et al., 2015).

Amylose is one of the key components that may influence the physiochemical properties and digestibility of rice. Rice varieties high in amylose content helped to reduce the level of glucose and slower the activity of the human gastrointestinal tract as compared to low‐amylose rice varieties (Denardin et al., 2012; Frei et al., 2003). Besides amylose content, several other factors that influence the physiochemical characteristics and digestibility of rice flour are processing techniques, rice varieties, structure, granule size, and their composition such as amylopectin, protein, and lipid contents (Lin et al., 2011; Yu et al., 2012; Zhu et al., 2011).

In this context, numerous studies have been conducted, but most of the work was done on Indica, Japonica, and Waxy varieties. Due to limitations, there is a need to study the physicochemical, structural properties, and digestibility of other rice varieties. In the present study, four varieties of rice flour (i.e., white rice, black rice, brown rice, and waxy rice) were selected to investigate its solubility, swelling power, and thermal and enzymatic hydrolysis properties.

## 2 MATERIALS AND METHODS

### 2.1 Reagents and raw materials

Four commercial brand rice varieties were purchased from the local market of Guangzhou, China. Black rice and brown rice were from Posh Brand, and white rice was from the Xiguyuanji brand, whereas, white waxy rice was from the Blue wheel brand. Megazyme assay kits were used to analyze total starch content (Megazyme International Ltd. Co.). All chemicals used for analysis were of analytical grade.

### 2.2 Rice flour formulation

All samples were ground into a powder with a hammer mill (Miller 6850). Then, the samples were passed through a sieve (100‐mesh). Samples were stored in a plastic bag before analysis.

### 2.3 Chemical composition analysis

The iodine colorimetric method was used to determine the amylose portion of ground samples with minor modifications (Ratnayake et al., 2001). The fat and protein content of rice flour samples were analyzed by adopting standard methods defined by AOAC (2002).

### 2.4 X‐ray diffraction (XRD)

The X‐ray diffractometer (40 KV, 40 mA) operating with Cu Kα radiation (*λ* = 0.154 nm) was used (Rigaku). All samples of flour taken for analysis were cooked at a temperature of 90°C for 30 min. Then, the cooked samples were placed in the freeze‐dryer for drying and further ground. Both the freeze‐dried and raw samples were tightly packed in a glass cell of rectangular shape and placed for scanning ranged between 4 and 35° 2θ angle at a rate of 2°/min (Shi et al., 2017). For relative crystallinity, peak Fit software was used and measured as crystalline peak area/total diffraction ratio (Systat Software Inc., Version 4.0).

### 2.5 Gelatinization properties

Sample of rice flour (3 mg, db) with 70%, w/w deionized water was scanned with the help of a differential scanning calorimeter (DSC‐8000, Perkin‐Elmer) at the temperature of 30 to 150°C at 10°C/min rate in a pan made up of stainless steel (Zhang et al., 2012). The sealed pan was left overnight to equilibrate the samples. Peak (*T*_p_), onset (*T*_o_), enthalpy of gelatinization (∆*H*), and conclusion (*T*_c_) were taken with the help of software provided with the DSC instrument (DSC‐8000, Perkin‐Elmer).

### 2.6 Pasting parameters

To make the total weight of 100 g (6% dry starch, w/w), each sample of flour (6 g, db) was put up with deionized water; then, a sample of accurate weight was analyzed by Brabender Viso‐ amylograph at 95°C (1.5°C/min) heat and after this was cooled to 50°C (1.5°C/min). Final viscosity (FV), peak viscosity (PV), and hot paste viscosity (HPV) were obtained from Brabender profiles. Breakdown (BD) and set back (SBV) viscosity were measured by software supplied with the instrument.

### 2.7 Swelling power (SP) and solubility (S)

Swelling power (SP) and solubility (S) were examined according to previous procedure with slight modifications (Adebooye & Singh, 2008; Li & Yeh, 2001). According to this method, about 500 mg sample was cooked with 20 ml water at 90°C for 30 min. Then, a solution was set to be cooled and centrifuged (2,600 *g*) for 15 min. The supernatant was gradually poured into a tube, and the resulted residue was measured to determine swelling power. The supernatant was shifted to glass and boiled for evaporation. Afterward, a sample was dried to a constant weight at 105°C temperature and weighed. Swelling power (SP) and solubility (S) were assessed by using the following equations.

(1) $$\text{Swelling}\;\text{power}\;\left(\text{SP}\right)=\frac{W_{t}}{\left(W‐W_{r}\right)}$$

(2) $$\text{Solubility}\;\left(S\right)=\frac{W_{r}}{W}\times 100\%$$

where *W*_t_: the weight of wet sediment; *W*_r_: the weight of dried supernatant; *W*: the weight of a sample.

### 2.8 Starch digestion

With little adaption in the previously used method, in vitro starch digestion was done (Butterworth et al., 2012). With phosphate saline buffer (15 ml), a sample of flour (∼50 mg, dry basis) was cooked at the temperature of 90°C for 30 min, and constant mixing was done and after that was placed for cooling to 37°C before the addition of enzyme (α‐amylase) solution (3.5 units). At each time interval up to 120 min, an aliquot (300 µl) was mixed with ice‐cold Na_2_CO_3_ solution (0.5 M, 1,200 µl) to stop unwanted reactions and centrifuged (4,000 *g*) for 5 min to remove an undigested portion of starch. Maltose equivalent level was determined by using PAHBAH assay (Para‐hydroxybenzoic acid hydrazide) (H9882, Sigma) (Moretti & Thorson, 2008). Maltose equivalent was calculated in percentage by using the following formula.

(3) $$\text{Maltose}\;\text{equivalent}\;\text{released}=\frac{\text{Total}\;\text{weight}\;\text{of}\;\text{equivalent}\;\text{maltose}\;\text{in}\;\text{supernatant}}{\text{Dry}\;\text{weight}\;\text{of}\;\text{starch}}\times 100\%$$

Kinetic profiles of starch digestion were fixed with a first‐order equation of Log of slope (LOS) analysis (Butterworth et al., 2012).

(4) $$\ln\;\left(\frac{\mathrm{dC}}{\mathrm{dt}}\right)=\ln\;\left(C_{\infty }k\right)‐kt$$

In this equation, digestion time in minutes was represented by *t* and *C* is the concentration of digested starch at the time of incubation *t*, *C*_∞_ is representative of digestion at time of infinity, and *K* is a constant rate (min^−1^). The plot of ln (*dC*/*dt*) against digestion time *t* is intellectually linear with a slope of −*k*, and the *C* can be calculated from the intercept of the equation and slope *k*.

### 2.9 Statistical analysis

Significance difference and mean value were analyzed by using the least significant difference (LSD) with the help of SPSS 18.0 statistical software (SPSS, Inc.). The significance level was .05.

## 3 RESULTS AND DISCUSSION

### 3.1 Chemical composition

The protein, fat, amylose, and total starch contents of rice flour are shown in Table 1. The protein content of flour samples ranged from 6.8% to 8.4% was found to be higher in black rice flour (8.4%) than white rice flour (7.9%), waxy rice flour (6.9%), and brown rice flour (6.8%). The obtained values were comparable to those previously conducted study (Dhital et al., 2015). Moreover, different factors such as cultivar, environmental conditions, and processing parameters significantly affect protein content (Dhital et al., 2015). The fat contents of all rice flour samples were ranged from 1.3% to 3.5% (Table 1), and the highest value was observed in brown and black rice (3.5% and 3.2% respectively). Physicochemical, rheological, and nutritional properties of starch‐based materials are significantly affected by amylose. In all rice flour, amylose contents were observed in a range from 3.0% to 21.8% (Table 1). The waxy rice flour showed significantly lower amylose content than brown, black, and white rice flour. Based on amylose content, rice is categorized as a high‐, intermediate‐, and low‐amylose rice. Black, brown, and white rice were considered as intermediate while waxy as low‐amylose rice. Results of amylose content are in line with previous findings (Dhital et al., 2015; Mir et al., 2013). Moreover, the amylose has affected the texture of cooked rice (Li et al., 2016). Total starch contents in rice flour samples were ranged from 68.1% to 85.3%, and flour from waxy rice contained more starch as compared to the other rice flours (Table 1). These results of total starch are consistent with the previous studies on rice flour (Dhital et al., 2015; Lu et al., 2009).

**TABLE 1:** Chemical composition of rice flours

Samples	Amylose (%)	Protein (%)	Fat (%)	Total starch (%)
Brown rice	20.5 ± 0.1^b^	6.8 ± 0.5^a^	3.5 ± 0.4^c^	79.1 ± 2.1^b^
Black rice	21.8 ± 0.4^c^	8.4 ± 0.3^b^	3.2 ± 0.3^c^	68.1 ± 0.2^a^
White rice	20.1 ± 0.6^b^	7.9 ± 0.2^b^	2.5 ± 0.1^b^	82.3 ± 0.6^c^
Waxy rice	3.0 ± 0.6^a^	6.9 ± 0.4^a^	1.3 ± 0.2^a^	85.3 ± 0.2^d^

Note

Data within the same column with the same letters are nonsignificant (*p* < .05).

### 3.2 X‐ray diffraction (XRD)

**FIGURE 1:**
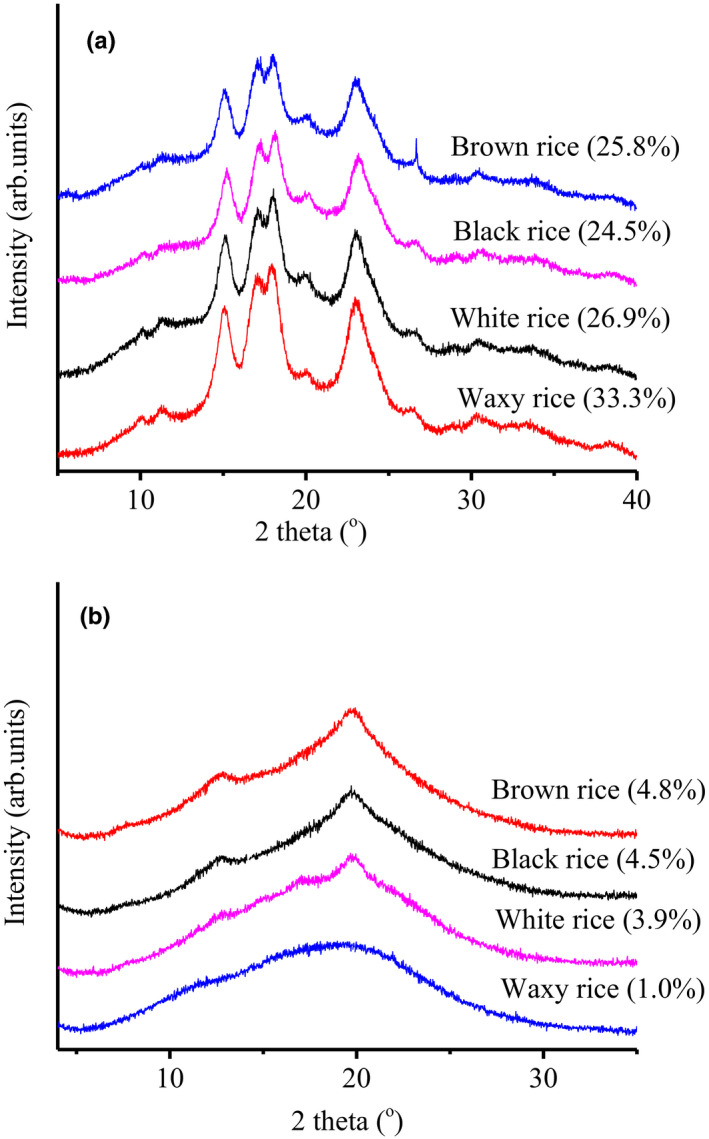
Relative crystallinity (%) and crystalline peaks of rice flours (a) raw rice flour (b). Freeze‐dried cooked sample

The native rice flour exhibited a clear A‐type diffraction pattern with a strong reflection peak at 2θ = 15.0° and 23.0° and an unresolved doublet at ca. 17.0° and 18.2° 2θ (Figure 1a). The results of this parameter are consistent with the previously stated report (Zhu et al., 2011), who reported the waxy rice (3.0%) and intermediate‐amylose rice (20.1%–21.8%) had the same A‐type diffraction pattern at 15.0°, unresolved doublet at 17.0° and 18.2°. The degree of relative crystallinity for all rice samples was varied from 24.5% to 33.3%. The waxy rice flour (33.3%) displayed higher crystallinity as compared to brown, black, and white rice flours (26.9%, 25.8%, and 24.5% respectively). The lower crystallinity value of black rice was due to the presence of naturally occurring pigments, that is, anthocyanin. Furthermore, it has been stated that lower crystallinity in rice is due to the higher amylose content (Chung et al., 2011). It has also been described that the presence of nonstarch components affected the structure and crystallinity of rice flours (Ibáñez et al., 2007).

Figure 1b shows the degree of crystallinity of cooked samples at 90°C for 30 min. It is evident from the figure that the crystallinity value of all rice flour samples was decreased by cooking. The lowest value was detected in the waxy rice flour sample (1.0%), whereas the highest value was observed in brown rice flour samples (4.8%). All flour samples were gelatinized after heating at 90°C and the typical A‐type diffraction patterns in all samples vanished. Also, as shown in Figure 1b, all X‐ray diffractograms in cooked rice samples displayed two weak peaks at 13 and 20°C that were more prominent as compared to raw flours and can be attributed to Vh‐type amylose–lipid complexes. These results were consistent with our previous study (Farooq et al., 2018). However, the pattern of cooked waxy flour displayed no peak for the amylose–lipid complex due to the lack of amylose content.

### 3.3 Gelatinization properties

Two distinct peaks were observed in all rice flour samples; peak I is attributed to the melting of the double helix while peak II indicates the complex formation between amylose and lipids. The onset (*T*_o_), peak (*T*_p_), and conclusion (*T*_c_) gelatinization temperatures and gelatinization enthalpy (∆*H*) varied significantly. The gelatinization temperatures of all rice flour were varied from 69.4 to 73.0°C for onset temperature; 76.0 to 81.4°C for peak temperature; and from 80.9 to 88.7°C for conclusion temperature. Brown rice flour displayed a higher onset temperature (*T*_o_ = 73.0°C) than black and white rice flours (71.0 and 72.3°C, respectively). It is evident from data that *T*_o_ peak increases with the increase in amylose content. Also, waxy rice flour displayed lower *T*_o_ temperature as compared to nonwaxy rice flours. The lower gelatinization temperature in waxy rice flour as compared to nonwaxy one was attributed to the more crystalline region and lower gelatinization temperature (Cooke & Gidley, 1992). Gelatinization parameters are also influenced by the size of granules, molecular structure (double‐helical structure), and crystallinity (Wang et al., 2010). The gelatinization enthalpy (∆*H*_g_) of waxy rice flour (9.9 J/g) was higher than brown, black, and white rice flours (6.9, 3.9, and 6.3 J/g). It is due to the lower gelatinization temperature, and enthalpy value increased with decreasing amylose content (Biliaderis et al., 1986). Moreover, the higher gelatinization temperature and lower enthalpy value in nonwaxy rice flour are also linked with lower swelling power and solubility (Table 2). Furthermore, an intact cell wall in brown and black rice flour can also be responsible for lowering the ∆*H* value by retarding the water movement in the starch granules.

**TABLE 2:** Swelling power (SP) and solubility (S) of rice flours

Samples	Swelling power (SP) (g/g)	Solubility (S) (%)
Brown rice	7.6 ± 0.5^b^	15.7 ± 0.3^b^
Black rice	6.6 ± 0.2^a^	13.5 ± 0.6^a^
White rice	8.9 ± 0.1^c^	17.6 ± 0.9^c^
Waxy rice	13.1 ± 1.1^d^	22.6 ± 1.0^d^

Note

Data within the same column with the same letters are nonsignificant (*p* < .05).

The second endotherm, corresponding to the dissociation of the amylose–lipid complex, was only detectable in brown, black, and white rice flours. Values of *T*_o_, *T*_p_, and *T*_c_ for the second endotherm were between 101.2 and 105.2°C, 104.4 and 111.0°C, and 106.3 and 113.3°C respectively, whereas no amylose–lipid complex peak was noticed in waxy rice flour due to lack of amylose. Brown and black rice flour displayed higher enthalpy values for the amylose–lipid complex dissociation peak as compared to white rice flours due to their relatively higher amylose content (Table 1).

### 3.4 Pasting parameters

**FIGURE 2:**
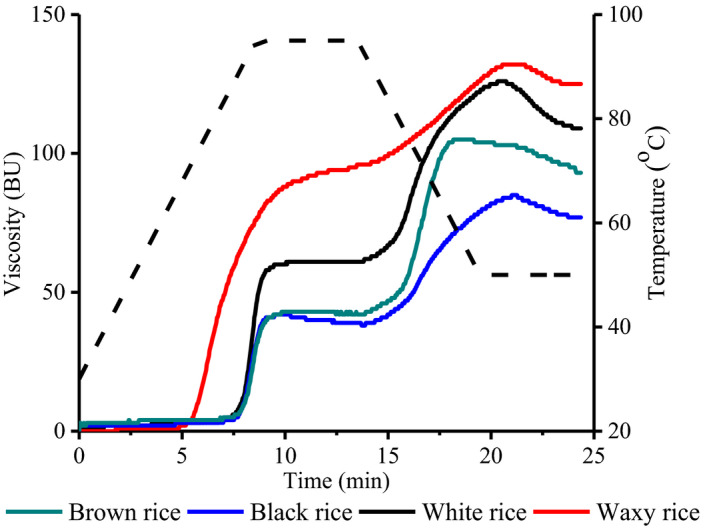
Pasting parameters of rice flours by using Brabendor‐Visco Amylo graph

The pasting parameters of rice flour samples are displayed in Table 3 and Figure 2. The pasting parameters of rice flour show its pasting performance during cooking and cooling. Peak viscosity (PV) reflects the swelling ability of starch during heating. Waxy rice flour exhibited higher PV (96 BU) (Table 3), which indicates that waxy rice flour requires less energy for cooking due to high‐amylopectin content and low‐amylose content. Black, brown, and white rice flour exhibited lower pasting and viscosity values. The differences in the pasting and viscosity values among the rice flour samples were because of the divergence in the content of amylose. Accordingly, black, brown, and white rice flour showed significantly low peak viscosity, hot paste viscosity (HPV), and final viscosity (FV); however, it provides higher setback viscosity (SBV). It was due to the presence of other nonstarch factors such as protein and lipids, which affected the pasting properties of rice flour (Dautant et al., 2007). In our study, the highest pasting viscosity and lower set back was observed in waxy rice flour. It has been reported that amylose content is negatively correlated with pasting viscosity and positively correlated with setback viscosity (Chao et al., 2014). The higher PV in waxy rice flour was attributed to the lower amylose content (Jane et al., 1999). Nonwaxy rice flour exhibited lower final viscosity (FV) and higher set back viscosity (SBV) as compared to waxy rice flour. It indicates the stability and retrogradation tendency of rice flour during storage. The pasting parameters of all rice flour are affected by the amylose content, amylopectin branch chain length, and other constituents. It has been postulated that higher swelling of granules is related to the amylopectin, whereas amylose and other constituents (e.g., lipids) restrict the granules to swell extensively (Mir & Bosco, 2014).

**TABLE 3:** Pasting parameters of rice flours by using Brabendor‐Visco Amylo Graph

Sample	PV (BU)	HPV	FV (BU)	SBV (BU)
Brown rice	43 ± 0.1^a^	42 ± 0.1^b^	104 ± 0.4^b^	63 ± 1.0^c^
Black rice	42 ± 1.2^ab^	38 ± 1.5^a^	79 ± 0.3^a^	40 ± 0.6^b^
White rice	61 ± 0.0^c^	61 ± 0.0^c^	122 ± 0.3^c^	61 ± 1.5^c^
Waxy rice	96 ± 0.0^d^	96 ± 0.0^d^	125 ± 0.3^d^	30 ± 0.5^a^

Note

Data within the same column with the same letters are nonsignificant (*p* < .05).

### 3.5 Swelling power (SP) and solubility (S)

Heating starch in presence of water results in the production of viscous paste that is utilized in many commercial applications. The swelling power (SP) of all rice flour was ranged from 6.6 to 13.9 g/g, which is in line with previous findings (Yu et al., 2012). According to these findings, it was stated that the swelling power of rice flour depends on the nonstarch components (i.e., proteins and lipids or channels in rice flour granules) (Yu et al., 2012). According to these findings, it was stated that swelling properties of cereals starches were significantly related to the amount of amylopectin. When rice starch was being gelatinized, the change in hydrogen bonding and molecular structure results in the leaching of amylose content from starch. So, swelling power is significantly related to both amylose and amylopectin content. Moreover, the amylose–lipid complex reduced the charged molecules and inhibit the swelling of rice flour (Falade & Christopher, 2015).

The waxy rice flour displayed a significantly higher solubility (S) value (22.6%) as compared to the black, brown, and white rice flours (13.5%, 15.7%, and 17.6%, respectively). The solubility value was found lower in rice with high‐amylose content than waxy rice flour due to its noneasily rupturing and more compact structure (Wani et al., 2012; Yu et al., 2012). Thus, the less compact structure of waxy rice flour displayed relatively higher swelling power and solubility values. Furthermore, different factors such as amylose‐to‐amylopectin ratio, granule structure, rice starch distribution in granules, protein, and lipid contents influence the swelling power and solubility (Reddy et al., 2016; Yu et al., 2012).

### 3.6 Starch digestion

The rate of starch digestion is enhanced by concentration, and type(s) of enzyme concentration and type(s) of an enzyme are responsible factors to speed up the rate of starch digestion (Warren et al., 2015). For example, both α‐amylase (endo‐acting) and amyloglucosidase (exo‐acting) exhibit antagonistic effects in the digestion of cooked starches (Zhang et al., 2013). Consequently, the α‐amylase enzyme was used to investigate the digestion rate of four rice varieties in cooked form. The α‐amylase activity conditions were set to attain a logarithmic digestion curve and to fit the first‐order kinetics, which illustrates the logarithmic curves for all flour samples (Butterworth et al., 2012). The α‐amylase activity depends on the nature of the starch substrate and the botanical origin of granules (Zhang et al., 2013).

**FIGURE 3:**
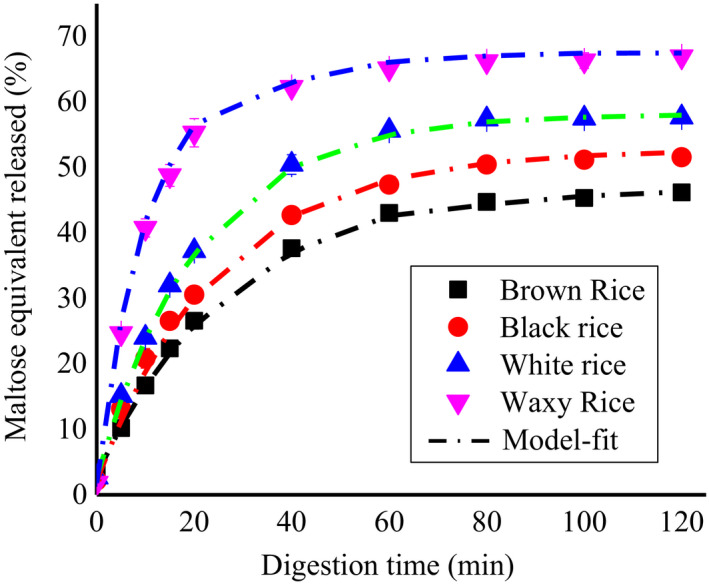
LOS digestion plots from cooked rice flours

As the first‐order fit of digestion progress curves shown in Figure 3, the single rate coefficients and the digestion extents after 2 hr of digestion are concise in Table 4. All rice flour samples showed a significant increase in the digestion rate after the first 30 min and a decrease in rate was observed after the extended time, whereas a lower rate of digestion was observed in nonwaxy rice flour samples. This is most likely because of the high‐amylose content present in rice flour samples.

**TABLE 4:** Mean values of digestion rate coefficient (*k*, min^−1^) and reducing sugar released extent after 2 hr of digestion of rice flour in cooked forms

Sample	*K* (min^−1^)	Maltose equivalent released (%)	*C*_∞_ (%)
Brown rice	0.036	46.1 ± 1.0^a^	47.9 ± 0.8^a^
Black rice	0.040	49.6 ± 0.8^b^	52.7 ± 0.4^b^
White rice	0.048	55.7 ± 0.4^c^	58.1 ± 0.1^c^
Waxy rice	0.058	63.9 ± 0.8^d^	67.5 ± 0.5^d^

Note

Data within the same column with the same letters are nonsignificant (*p* < .05).

Brown rice and black rice flour showed a relatively low enzymatic digestion rate (Figure 3) as compared to white rice flour. The reason behind this is that the outer intact layer of brown rice and black rice reduces the enzymatic susceptibility of the enzyme. Different rice cultivars may have variations regarding the nature of starch, protein, and dietary fiber, so any change in their structure may influence the starch digestibility (An et al., 2016). Protein reduced starch digestibility by limiting its rate of swelling and gelatinization (Klunklin & Savage, 2018). Black rice showed a significant decrease in starch digestibility due to having high protein contents. According to previous studies, black and purple rice showed lower digestibility due to the presence of phenolics (An et al., 2016; Klunklin & Savage, 2018). Moreover, enzymatic digestion of rice flour is also exaggerated by other factors like particle size, crystalline structure, surface pores, the degree of polymerization (DP), nonstarch components (e.g., proteins, fats, ash, and fibers), interactions of nonstarch with starch components, and amylose–amylopectin ratio (Mahasukhonthachat et al., 2010). When four rice varieties were compared, waxy rice flour samples gave higher *k* values and released more reducing sugars than brown, black, and white rice flours samples, representing that waxy rice showed fast digestion as compared to nonwaxy rice. This is probably related to their higher swelling power, solubility (Table 5), and higher pasting properties (Table 2) of waxy rice flour, as compared to nonwaxy rice flour, which makes it more susceptible to enzymatic digestion.

**TABLE 5:** Gelatinization properties of rice flours by using differential scanning calorimeter (DSC)

Samples	Peak I				Peak II			
	*T*_o_ (^o^C)	*T*_p_ (^o^C)	*T*_c_ (^o^C)	∆*H* (J/g)	*T*_o_ (^o^C)	*T*_p_ (^o^C)	*T*_c_ (^o^C)	∆*H* (J/g)
Brown rice	73.0 ± 0.2^d^	76.0 ± 0.5^a^	85.8 ± 1.2^b^	6.3 ± 0.2^b^	103.0 ± 1.1^ab^	107.0 ± 1.7^b^	110.9 ± 1.5^b^	1.3 ± 0.3^b^
Black rice	71.0 ± 0.1^b^	76.3 ± 0.3^a^	80.9 ± 0.5^a^	3.9 ± 0.1^a^	105.2 ± 1.5^b^	111.0 ± 1.4^c^	113.3 ± 1.1^c^	1.0 ± 0.0^b^
White rice	72.3 ± 0.2^c^	77.9 ± 1.0^b^	87.1 ± 1.6^b^	6.9 ± 0.3^c^	101.2 ± 1.5^a^	104.3 ± 0.4^a^	106.3 ± 0.6^a^	0.5 ± 0.2^a^
Waxy rice	69.4 ± 0.4^a^	81.4 ± 1.0^c^	88.7 ± 1.2^bc^	9.9 ± 0.9^d^	ND	ND	ND	ND

Note

Data within the same column with the same letters are nonsignificant (*p* < .05).

Abbreviation: ND, not detected.

## 4 CONCLUSIONS

Differences were found in the chemical composition, physicochemical and functional properties, and digestibility of four rice varieties. The research indicated that black rice had higher amylose, protein, and fat content along with lower starch content as compared to the brown, white, and waxy rice flour. Brown, black, and white rice had higher *T*_o_ and *T*_c_ and lower ∆*H* that may be due to higher amylose content than waxy rice flour. All rice flour exhibited an A‐type X‐ray diffraction pattern. Black and brown rice showed significantly lower digestibility than white and waxy rice flour. This study revealed that brown and black rice flour could be an effective alternative in different food formulations due to their low starch digestibility, low swelling power and solubility, high‐amylose content, and higher amount of nonstarch components.

## CONFLICT OF INTEREST

All authors declare that they have no conflict of interest.
